# Thermal Liquid Biopsy: A Promising Tool for the Differential Diagnosis of Pancreatic Cystic Lesions and Malignancy Detection

**DOI:** 10.3390/cancers16234024

**Published:** 2024-11-30

**Authors:** Judith Millastre, Sonia Hermoso-Durán, María Ortiz de Solórzano, Nicolas Fraunhoffer, Guillermo García-Rayado, Sonia Vega, Luis Bujanda, Carlos Sostres, Ángel Lanas, Adrián Velázquez-Campoy, Olga Abian

**Affiliations:** 1 Service of Digestive Diseases, University Clinic Hospital Lozano Blesa, 50009 Zaragoza, Spain; millastrej@gmail.com (J.M.); ortizdsm6@gmail.com (M.O.d.S.); guillermogarcia7@hotmail.com (G.G.-R.); carlossostres@gmail.com (C.S.); alanas@unizar.es (Á.L.); 2 Instituto de Investigación Sanitaria Aragón (IIS Aragón), 50009 Zaragoza, Spain; shermosod@gmail.com; 3 Centro de Investigación Biomédica en Red en el Área Temática de Enfermedades Hepáticas y Digestivas (CIBERehd), 28029 Madrid, Spain; luis.bujandafernandezdepierola@osakidetza.eus; 4 Programa Franco-Argentino de Estudio del Cáncer de Páncreas, Buenos Aires, Argentina; nicolasfraunhoffernavarro@gmail.com; 5 Centre de Recherche en Cancérologie de Marseille (CRCM), INSERM, CNRS UMR, Aix-Marseille Université, 13009 Marseille, France; 6 Institut Paoli-Calmettes, Parc Scientifique et Technologique de Luminy, Equipe Labellisée La Ligue, 13288 Marseille, France; 7 Institute for Biocomputation and Physics of Complex Systems, University of Zaragoza, 50018 Zaragoza, Spain; svega@bifi.es; 8 Donostia University Hospital, University of the Basque Country (UPV/EHU), 20014 San Sebastian, Spain; 9 Department of Biochemistry and Molecular and Cell Biology, University of Zaragoza, 50009 Zaragoza, Spain

**Keywords:** differential diagnosis, early diagnosis, intracystic fluid, machine learning, mucinous cysts, non-mucinous cysts, pancreatic cancer, pancreatic cystic lesions, pancreatic ductal adenocarcinoma, thermal liquid biopsy

## Abstract

**Background/Objectives**: Mucinous epithelial pancreatic cystic lesions (PCLs) are premalignant lesions readily detectable through imaging techniques such as multidetector computed tomography, magnetic resonance imaging, and endoscopic ultrasound (EUS). However, distinguishing these from other PCLs with lower or no malignant potential, and the early identification of those undergoing malignant transformation, remains a diagnostic challenge. Current methods, including biochemical markers in intracystic fluid (ICF) and genomic studies, offer some assistance but are not always reliable or accessible. Thermal liquid biopsy (TLB) is a novel diagnostic tool that examines the thermal profile (thermogram) of biological samples, reflecting their response to heat and thereby revealing characteristics of their overall composition or disease-induced alterations. **Methods**: In this retrospective study, a total of 35 ICF samples (49% mucinous) obtained via EUS-FNA (fine needle aspiration) were analyzed using TLB. Thermogram data were utilized to develop predictive models for differential diagnosis between mucinous and non-mucinous PCLs or malignancy detection through machine learning algorithms. **Results**: Two classification models were developed: TLB1 (“mucinous” vs. “non-mucinous” PCLs) and TLB2 (“benign mucinous” vs. “malignant mucinous” PCLs). The TLB1 model demonstrated a sensitivity of 92% and a negative predictive value of 86%, with an area under the curve (AUC) of 0.79 (0.59–0.99), indicating good discriminative ability between the two groups. The TLB2 model exhibited excellent predictive capability, with an AUC of 1.00. **Conclusions**: TLB analysis of PCLs is a promising tool that could significantly enhance the differential diagnosis of PCLs, enabling the efficient identification of mucinous lesions and even those undergoing malignant transformation.

## Simple Summary

Mucinous epithelial pancreatic cystic lesions (PCLs) are premalignant lesions detectable through imaging techniques; however, distinguishing them from other PCLs with lower malignancy potential is challenging. Current methods like biochemical markers and genomic studies are not always reliable. Thermal liquid biopsy (TLB) is an innovative tool that analyzes the thermal profile of biological samples to detect disease-related alterations. In a retrospective study of 35 intracystic fluid samples obtained via fine needle aspiration, predictive models were developed using machine learning algorithms. Two classification models were created: TLB1, which differentiates mucinous from non-mucinous PCLs, demonstrating 92% sensitivity and 86% negative predictive value, and TLB2, which identifies benign and malignant mucinous lesions, achieving an area under the curve of 1.00. TLB shows promise in improving the differential diagnosis of PCLs and in detecting malignant transformations.

## 1. Introduction

Pancreatic cystic lesions (PCLs) are a heterogeneous group of lesions with increasing incidence, usually identified incidentally on imaging studies (multidetector computed tomography (MDCT), magnetic resonance imaging (MRI), or endoscopic ultrasound (EUS)). Their clinical relevance lies in the potential of mucinous subtypes like mucinous cystic neoplasms (MCNs) or intraductal papillary mucinous neoplasms (IPMNs) to develop into pancreatic ductal adenocarcinoma (PDAC), which is currently a major health problem due to its increasing incidence and poor prognosis, with surgery being the only potentially curative treatment [1].

Accurate characterization of mucinous PCLs (M-PCLs) and early detection of malignancy are crucial for effective management of this at-risk population. The current diagnostic approach is based on a combined analysis of the clinical context, imaging tests, and biochemical markers or genomics. The accuracy or availability of these methods is limited, and pancreatic surgery is burdened by high morbimortality, especially in the cephalic duodenopancreatectomy (CDP) [2]. Novel diagnostic modalities are highly desirable, especially those that are ideally minimally invasive, cost-effective, and accessible, to avoid unnecessary risks and reduce costs from unnecessary follow-ups.

Thermal liquid biopsy (TLB) is the application of differential scanning calorimetry (DSC) to biological liquid samples. DSC is a calorimetric technique that measures the excess heat capacity (C_P_) associated with biomolecular transitions as a function of temperature. It provides thermal denaturation profiles (thermograms) that reflect the behavior and interaction of metabolites when heat is applied, providing global insight into the composition of the sample [3]. This method is expanding in the biomedical field, analyzing several types of samples [4,5,6,7], demonstrating its diagnostic potential.

Our group has been studying TLB as a complementary tool in different pathologies [8,9], proposing methodologies to analyze thermograms [10,11]. With the advent of artificial intelligence, the group has recently proposed a methodology to obtain classification models based on thermograms from serum samples [12].

Intracystic fluid (ICF) is a biological fluid composed of proteins and other metabolites. Its composition varies in every subtype of PCLs, reflecting their different pathophysiology. Our group has previously shown the feasibility of obtaining thermograms from ICF samples (ICF thermograms) [13]. Using more sophisticated tools to build machine learning models, we hypothesize that ICF-TLB can improve the differential diagnosis between M-PCLs and non-mucinous PCLs (NM-PCLs) and potentially detect early malignancy-induced changes. This approach could improve clinical decisions, optimizing patient outcomes.

## 2. Materials and Methods

Between January 2016 and May 2023, prospective ICF samples of PCLs with indication for EUS–fine needle aspiration (FNA) (>2 cm and/or presence of Worrisome Features (WF)/High-Risk Stigmata (HRS)) were collected from patients recruited at the University Clinic Hospital (Zaragoza), with a long interruption caused by COVID-19 (January 2020 to April 2021). The EUS-FNA procedure was performed with an Olympus^®^ 140 curvilinear echo-endoscope (Olympus, Barcelona, Spain). Boston Scientific^TM^ Expect^®^ 19 or 22-gauge needles were used depending on the cystic endosonographic features. For ethical reasons, DSC analysis was only carried out in the case of ICF remaining after analysis of biochemical markers, for which at least 1 mL Eppendorf was necessary for each patient.

All patients recruited were over 18 years old and had an indication for EUS-FNA regardless of their participation in the study, which was free and voluntary after receiving the relevant verbal and written information and signing the informed consent form. The procedure was approved by the Ethics Committee (Aragon Research Ethics Committee (CEICA) on 5 October 2016, Act No. 17/2016 and 5 February 2020, Act No. 03/2020.

Exclusion criteria: age under 18, diagnosis other than PCLs, insufficient ICF sample (<1 mL), inability to obtain a thermogram or to establish a diagnosis due to lack of clinical information.

Data collected: age, sex, smoking/alcohol habits, location (head or body-tail), size, MRI/EUS characteristics (WF/HRS, pathognomonic image (serous cystadenoma (SCA)), Wirsung communication, history of pancreatitis, related symptoms (diabetes, abdominal pain, jaundice, weight loss), string sign, ICF biomarkers (CEA (carcinoembryonic antigen) > 192 ng/mL and glucose < 50 mg/dL: higher probability of M-PCLs; amylase > 250 U/L: Wirsung communication), serum biomarkers (carbohydrate antigen 19.9 (Ca19.9) > 37 U/mL: malignancy if no cholestasis; bilirubin > 1.2 mg/dL: HRS), surgical resection, postoperative complications (Clavien–Dindo classification) [14], histopathological analysis, clinical evolution.

Diagnosis was established as *confirmed* (histopathologically or pathognomonic morphology in the case of SCA lesions) or *high probability* (consensus of three expert pancreatologists (radiologist, clinician, and endoscopist) based on a combined analysis of the clinical context, imaging tests, and biochemical markers).

The classification of PCLs is shown in Table 1.

### 2.1. Sample Processing

After EUS-FNA, ICF samples were frozen (−80 °C) and anonymized with internal codes following the recommendations of the local ethics committee. For the DSC assay, samples were thawed at room temperature, centrifuged, and diluted 1:10 in phosphate-buffered saline, using 400 μL to obtain the thermograms.

A high-sensitivity automated VP-DSC (MicroCal, Malvern Panalytical, Malvern, UK) was used. The scanning rate was 1 °C/min, with a scanning range between 10 and 95 °C.

### 2.2. Data Analysis

#### 2.2.1. Preparation of Thermograms

A software developed by the group, implemented in Origin 7.0 (OriginLab, Northampton, MA, USA), was used. It involved baseline subtraction and correction, interpolation to obtain uniformly distributed data across the temperature range (ΔT = 0.25 °C), and restricting the analysis to the interval between 40 and 95 °C. Each thermogram was normalized by its area. The temperatures and their excess CP values were the predictive variables for the classification model. To reduce the number of predictive variables and avoid overfitting, information was selected from each degree of temperature, truncating between 55 and 85 °C, as these values exhibited the most significant changes.

#### 2.2.2. Obtaining Classification Models (iTLB Models)

From the ICF thermograms, two iTLB models were obtained:

- iTLB1 Model: NM-PCLs vs. M-PCLs, training with confirmed LQP diagnoses and validating with high probability diagnoses.
- iTLB2 Model: benign (bM-PCLs) vs. malignant (mM-PCLs), training with all M-PCLs due to the small sample size.

The proposed thermogram analysis methodology was developed previously [12] and is based on obtaining a classification model from the shape information of the thermograms using pairs of temperatures through the K-Top-Scoring-Pair function (*R switchBox* library). The classification model was fitted using Lasso logistic regression by applying the *ncvreg* package in R (version 4.3.2) and cross-validated for model fit.

The classification model provided a single number per patient (−∞, +∞). If the model provided a number < 0, the lesion was classified as an NM-PCL (for iTLB1) or bM-PCL (for iTLB2), whereas if the number was >0, the lesion was classified as an M-PCL (for iTLB1) or mM-PCL (for iTLB2).

The performance of both models was evaluated by calculating common performance indexes: sensitivity, specificity, positive and negative predictive values (PPV and NPV), receiver operating characteristic (ROC) curve, and its area under the curve (AUC) with its 95% confidence interval (CI).

#### 2.2.3. Statistical Analysis

The Kolmogorov–Smirnov–Lilliefors test or the Shapiro–Wilk test (depending on the sample size) was used to assess the normality of the variables. Medians between two independent groups were compared using the Wilcoxon test for variables with non-normal behavior.

For all tests, a two-sided *p*-value < 0.05 was considered statistically significant. Statistical analyses were performed using the R language and environment for statistical computing, version 4.3.2 (31 October 2023).

## 3. Results

A total of 41 patients with PCLs were recruited; six met the exclusion criteria (insufficient information in the clinical history (n = 1); diagnosis other than PCL (n = 2: adrenal gland pheochromocytoma and pancreatic metastasis of primary renal tumor); incorrect nomenclature that prevented adequate identification of the sample with the patient (n = 2); and technical impossibility of obtaining the thermogram (n = 1)); thus, the final sample size was 35, of which 22 (63%) had pathological confirmation: 17 (49%) were M-PCLs, with 8/17 (47%) being mM-PCLs.

### 3.1. Demographics

Table 2 provides a summary of the cohort demographics. Tables S1 and S2 show more detailed characteristics.

### 3.2. Symptoms

Most PCLs (69%) presented symptoms:

- NM-PCLs: 67% (12/18) abdominal pain (2/5 SCAs and 10/11 PCs, nine with underlying chronic pancreatitis).
- M-PCLs: 71% (12/17) symptomatic.
  ○ bM-PCLs: 44% (4/9) abdominal pain, one of them (IPMN) with acute pancreatitis.
  ○ mM-PCLs: jaundice (100% of malignant IPMNs (mIPMNs) and 25% of PDACs) and weight loss (100% of PDACs and 67% of mIPMNs) were predominant.

Five patients with PCLs presented new-onset diabetes: two SCAs (both ≥40 mm) and three mM-PCLs (two mIPMNs and one PDAC).

### 3.3. Morphological Characteristics, String Sign, and Biochemical Markers

Table 3 summarizes the diagnostic performance of imaging tests, string sign, and biochemical markers.

Regarding HRS,

- Mural nodules > 5 mm (n = 3): two IPMNs (one mIPMN), one PC (disappeared in follow-up).
- Solid component (n = 4): All malignant, two PDACs, two mIPMNs.
- Dilated Wirsung > 10 mm: one main duct IPMN (MD-IPMN), not fit for surgery and negative cytology, favorable evolution to date.

Regarding WF,

- Cyst wall thickening (n = 4): two PCs, one SCA, one PDAC.
- Size > 30 mm: 6/8 M-PCLs.
- Dilated Wirsung 5–9 mm: one PDAC, five IPMNs (three mIPMNs).
- Lymphadenopathies: two PDACs.

Regarding biochemical markers, it should be noted that in the cohort, ICF glucose was available for only 10 patients.

### 3.4. Cytology

Performed in 43% of the samples: 20% hypocellular and 33% misdiagnosed (three malignancies and two mucinous changes misidentified).

### 3.5. Surgical Resection

Performed in 49% (17/35) of cases:

- Six distal pancreatectomies: one due to complication (pancreatic fistula), the remaining five due to suspected mM-PCLs with HRS/WF: pain (3/5), size > 30 mm (4/5), growth (3/5), acute pancreatitis (1/5), 8 mm mural nodule (1/5), elevated serum Ca19.9 (1/5). All of them were confirmed as M-PCLs, but only one showed high-grade dysplasia (HGD), with its only WF being growth. All of them presented minor complications.
- Five CDPs: All performed due to suspected mM-PCLs with HRS/WF: new-onset diabetes (5/5), jaundice (2/5), elevated serum Ca19.9 (3/5), size > 30 mm (3/5), dilated Wirsung (3/5), or solid component (2/5). Among these, two were confirmed as SCAs, with no postoperative complications, and three were confirmed as mM-PCLs, with one postoperative death.
- Three exploratory laparotomies, two cystogastrostomies, and one emergency surgery (hemoperitoneum).

### 3.6. ICF Thermograms

**Figure 1:**
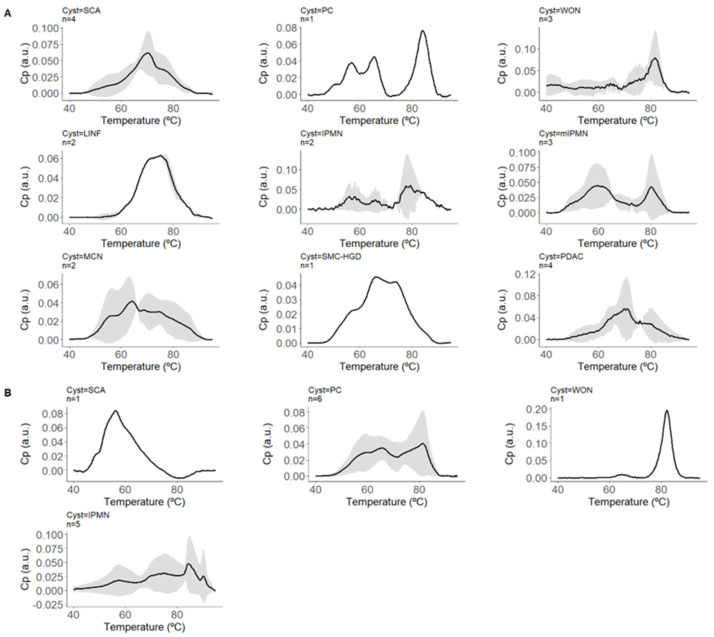
Normalized thermograms of intracystic fluid (ICF) for each pancreatic cystic lesion (PCL) subtype. This figure displays the means and standard deviations of the normalized thermograms of ICF for various PCL subtypes. The thermograms represent the excess heat capacity (C_P_) in arbitrary units (a.u.) as a function of temperature (°C), showing the variability both between and within individuals with each subtype. The black lines represent the mean C_P_ values, while the shaded areas indicate the standard deviations, illustrating the range of thermal responses for each subtype of PCL. This graphical representation helps visualize the distinct thermal profiles associated with each type of cystic lesion. Panel (**A**) represents cysts with confirmed diagnoses and includes thermograms for Serous Cystadenoma (SCA, n = 4), Pseudocyst (PC, n = 1), Walled-off Necrosis (WON, n = 3), Lymphoepithelial cyst/Lymphangioma (LINF, n = 2), Intraductal Papillary Mucinous Neoplasm (IPMN, n = 2), Mucinous Cystic Neoplasm (MCN, n = 2), Simple Mucinous Cyst with High-Grade Dysplasia (SMC-HGD, n = 1), and Pancreatic Ductal Adenocarcinoma (PDAC, n = 4). Panel (**B**) represents cysts with highly probable diagnoses and includes thermograms for Serous Cystadenoma (SCA, n = 1), Pseudocyst (PC, n = 6), Intraductal Papillary Mucinous Neoplasm (IPMN, n = 5), and Walled-off Necrosis (WON, n = 1).

The means and standard deviations of the normalized ICF thermograms for each PCL subtype were represented (Figure 1) to observe inter- and intra-individual variability.

#### 3.6.1. iTLB1 Model: NM-PCLs vs. M-PCLs

**Figure 2:**
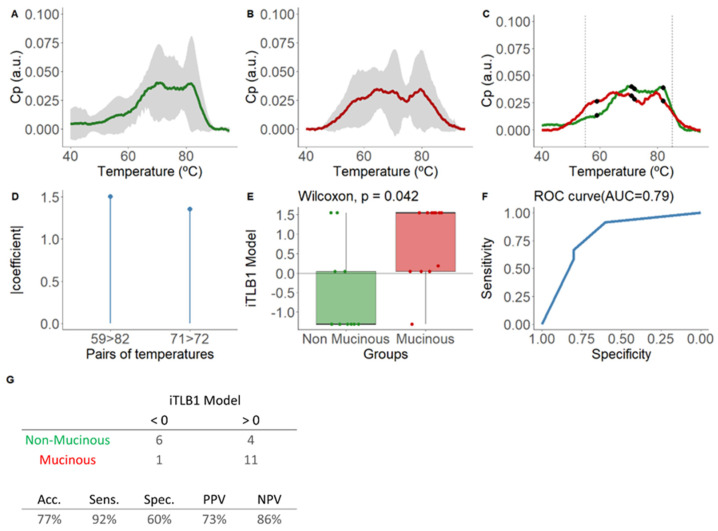
Mean normalized thermograms by area for each group and results of the iTLB1 model for differentiating thermograms of intracystic fluid from non-mucinous and mucinous lesions from PCLs with confirmed diagnosis. In panel (**A**), the green line represents non-mucinous PCLs, while in panel (**B**), the red line represents mucinous PCLs. The grey shading indicates the mean ± standard deviation for each group. Panel (**C**) displays the mean normalized thermograms by area for each group overlapping, with non-mucinous lesions in green and mucinous lesions in red. Black dots represent the temperatures used in the iTLB1 model, with vertical dashed lines marking the temperature range used to train the model (55–85 °C). After, this figure presents the results of the iTLB model obtained for differentiating the thermograms of intracystic fluid (ICF) from non-mucinous lesions (NM-PCLs) and mucinous lesions (M-PCLs). Panel (**D**) shows the absolute value of the coefficients of the iTLB1 model for each of the predictive temperature pairs. Panel (**E**) illustrates the median differences in the iTLB1 model results for each group (non-mucinous in green and mucinous in red), with the horizontal line representing the zero cutoff point. Panel (**F**) shows the area under the ROC curve (AUC) of the iTLB1 model. Panel (**G**) presents the contingency table for the prediction results (top) and the performance metrics of the iTLB1 model (bottom). Notes: C_P_: excess heat capacity; a.u.: arbitrary units; AUC: area under the curve; iTLB: intelligence Thermal Liquid Biopsy; Acc: accuracy; Sens: sensitivity; Spec: specificity; PPV: positive predictive value; NPV: negative predictive value.

Developed from the area-normalized ICF thermograms from PCLs with confirmed diagnoses, with 45% (10/22) being NM-PCLs (four SCAs, two lymphoepithelial/lymphangiomas (LINF), and four PCs), and 55% (12/22) being M-PCLs (five IPMNs (three mIPMNs), two MCNs, four pancreatic ductal adenocarcinomas with associated cystic component (cPDAC), and one simple mucinous cyst with HGD (SMC-HGD)). Figure 2A–C display their means and standard deviations.

The iTLB1 model consists of two temperature pairs, illustrated in Figure 2D, along with the absolute value coefficients assigned to each pair and depicted in the mean thermograms of each group in Figure 2C. Statistically significant differences were observed in iTLB1 scores between the medians of the NM-PCL group (−1.31 [−1.31;0.04]) and the M-PCL group (1.54 [0.04;1.54]) (Wilcoxon test: *p*-value = 0.042), with an AUC of 0.79 (95% CI = 0.59–0.99) (Figure 2E,F). The prediction results showed sensitivity, specificity, PPV, and NPV of 92%, 60%, 73%, and 86%, respectively (Figure 2G). In other words, 6/10 NM-PCLs and 11/12 M-PCLs were correctly classified.

**Figure 3:**
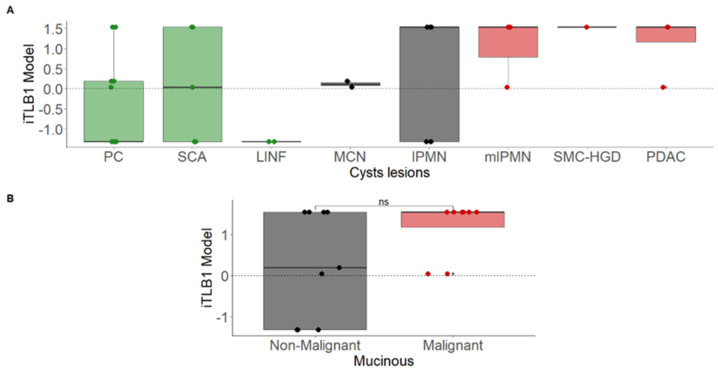
iTLB1 model scores for different cystic lesions in patients with confirmed and highly probable diagnoses. Panel (**A**) presents the scores by cyst type (non-mucinous in green, non-malignant mucinous in black, and malignant mucinous in red). Panel (**B**) shows the scores based on malignancy (red for malignant and black for non-malignant mucinous lesions). Notes: iTLB: intelligence Thermal Liquid Biopsy; PC: Pseudocyst; SCA: Serous Cystadenoma; LINF: Lymphoepithelial/Lymphangioma; MCN: Mucinous Cystic Neoplasm; IPMN: Intraductal Papillary Mucinous Neoplasm; mIPMN: malignant Intraductal Papillary Mucinous Neoplasm; SMC-HGD: Simple Mucinous Cyst with High-Grade Dysplasia; PDAC: Pancreatic Ductal Adenocarcinoma; ns: not significant.

Subsequently, the iTLB1 model was applied to the ICF thermograms of PCLs with high probable diagnosis (remaining 37%). Figure 3A shows the iTLB1 model scores for each type of lesion, considering all PCLs. Among the NM-PCLs, the misclassified cases were PC (5/11) and SCA (3/5). Among the M-PCLs, only 3/10 IPMNs were misclassified, all of them benign; thus, all mM-PCLs were correctly classified as mucinous. However, no statistically significant differences were observed between bM-PCLs (0.189 [−1.313;1.544]) and mM-PCLs (1.544 [1.169;1.544], Wilcoxon test: *p*-value = 0.180) (Figure 3B).

#### 3.6.2. iTLB2 Model: bM-PCLs vs. mM-PCLs

Developed to detect malignancy, given the ineffectiveness shown by the iTLB1 model. All M-PCLs (n = 17, 71% with confirmed diagnosis) were used for the training, with 53% being bM-PCLs (seven IPMNs, two MCNs) and 47% being mM-PCLs (three mIPMNs, one SMC-HGD, four PDACs).

**Figure 4:**
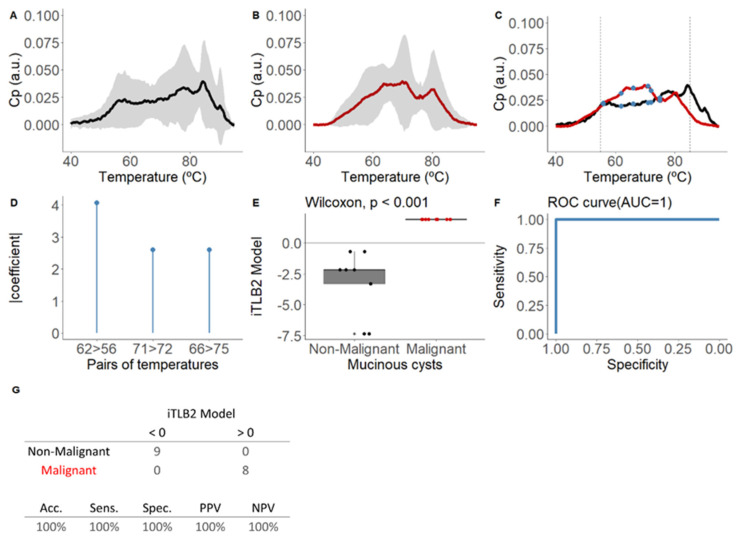
Mean area-normalized thermograms for mucinous group and results of the iTLB2 model for differentiating thermograms of intracystic fluid from benign mucinous and malignant mucinous lesions. In panel (**A**), the black line represents benign mucinous pancreatic cystic lesions (bM-PCLs), while in panel (**B**), the red line represents malignant mucinous pancreatic cystic lesions (mM-PCLs). The grey shading indicates the mean ± standard deviation for each group. In panel (**C**), the mean thermograms of both groups are superimposed, with vertical dashed lines marking the temperature range used to train the classification model (55–85 °C) and blue dots representing the temperatures used in the iTLB2 model. After, this figure presents the results of the iTLB2 model obtained for differentiating the thermograms of intracystic fluid (ICF) from benign mucinous lesions (bM-PCLs) and malignant mucinous lesions (mM-PCLs). Panel (**D**) shows the absolute value of the coefficients of the iTLB2 model for each of the predictive temperature pairs. Panel (**E**) illustrates the median differences in the iTLB2 model results for each group (benign mucinous in black and malignant mucinous in red), with the horizontal line representing the zero cutoff point. Panel (**F**) shows the area under the ROC curve (AUC) of the iTLB2 model. Panel (**G**) presents the contingency table for the prediction results (top) and the performance metrics of the iTLB2 model (bottom). Notes: CP: excess heat capacity; a.u.: arbitrary units; AUC: area under the curve; iTLB: intelligence thermal liquid biopsy; Acc: accuracy; Sens: sensitivity; Spec: specificity; PPV: positive predictive value; NPV: negative predictive value; bM-PCL: benign mucinous pancreatic cystic lesion; mM-PCL: malignant mucinous pancreatic cystic lesion.

Figure 4A–C show the means and standard deviations of the area-normalized ICF thermograms for each group.

The iTLB2 model consists of three temperature pairs, illustrated in Figure 4D, along with the absolute value coefficients assigned to each pair and depicted in the mean thermograms of each group in Figure 4C. Statistically significant differences were observed in the iTLB2 score between the medians of the bM-PCL group (−2.17 [−3.30;−2.17]) and the mM-PCL group (1.90 [1.90;1.90]) (Wilcoxon test: *p*-value < 0.001), with an AUC of 1.00 (95% CI = 1.00-1.00) (Figure 4E,F). The prediction results showed a performance index of 100% (Figure 4G).

## 4. Discussion

PDAC is the third leading cause of cancer deaths in the USA [15], with 20% of patients being surgical candidates at diagnosis, and a 5-year overall survival rate of 10% [16]. Its indolent course often leads to delayed diagnosis, exacerbated by the lack of effective biomarkers and screening programs [1]. The accurate identification of certain at-risk populations [17], including patients with M-PCLs [18], is crucial.

PCLs are a heterogeneous group of lesions with increasing incidence [19], ranging from inflammatory fluid accumulation residual to pancreatic diseases to true cysts with epithelial lining, which determines their malignancy risk. Within this group, M-PCLs, with their characteristic mucin-producing columnar epithelia, stand out. However, differentiating them from imaging tests is one of the current diagnostic challenges, especially when they are smaller than 2 cm, and other tools, such as ICF biomarkers or genomics, are not always accurate or available. Therapeutic decision-making should always be done in multidisciplinary committees, relying on clinical practice guidelines that, however, present a certain variability in their recommendations [20].

According to the series, M-PCLs were the most common epithelial cysts, with IPMNs (70%) predominating over MCNs (25%). SCAs account for 10–16% of total PCLs, and lymphoepithelial lesions/lymphangiomas are rare (0.5%). The study cohort mimics these reported frequencies, as well as data on distribution by sex, age, and location, as shown in Table 2. In surgical series, the most resected PCLs were also M-PCLs (45% IPMNs, 16% MCNs), followed by SCAs (16%) [21]. In this study, IPMNs were also the most frequently operated on (44%), followed by MCNs (12%) and SCAs (9%).

The sample size is small, and this is probably the biggest weakness of our study, together with its implementation in a single center. For this reason, this study is considered a pilot study, and we are already working on expanding the sample size in collaboration with other centers for future studies. However, despite the small size, it is a balanced sample with representation of the main subtypes, including rare ones such as LINF and SMC [22]. The predominance of symptomatic patients is explained by the inclusion of PCs on underlying chronic pancreatitis and the selection biases inherent to the exclusive recruitment of patients with PCLs using EUS-FNA criteria, including symptomatic PCLs.

The methodology applied to obtain iTLB models was previously described in serum samples [12]. However, there was a difference when it was applied to ICF thermograms. A range temperature between 60 and 80 °C was used in serum samples, while a bigger range was applied in ICF samples (55–85 °C). The reason for this change is that ICF samples experience less cooperative unfolding between their proteins, leading to thermograms whose C_P_ value starts to increase at lower temperatures and ends at its baseline at higher temperatures than serum samples. This phenomenon can be observed by comparing the thermogram figures in this manuscript with, for example, the article published on serum samples [12].

### 4.1. Differential Diagnosis of NM-PCLs vs. M-PCLs

In clinical practice, the diagnostic workup begins with imaging tests, with a reported accuracy of diagnosis of 47–78% with most advanced modalities [23]. In a recent meta-analysis [24], MDCT showed an accuracy of 39–45%, and EUS, limited by interobserver variability, had a sensitivity and specificity of 36–91% and 45–81% [25]. In the study cohort, the accuracies of MDCT and MRI were 75% and 56%, respectively, and EUS had a sensitivity and specificity of 67% and 90%.

String sign showed a sensitivity, specificity, PPV, and NPV for M-PCLs of 59%, 83%, 77%, and 68%, similar to the study [26].

Regarding ICF biomarkers, in a recent meta-analysis, CEA showed a sensitivity and specificity for M-PCLs of 58% and 87% [27]. This study cohort had a higher sensitivity (76%) and slightly lower specificity (82%). However, it is reported that up to 30% of IPMNs have CEA levels < 192 ng/mL, and in this study, it was observed in 20% of IPMNs and 50% of MCNs. Additionally, CEA is not valid for distinguishing IPMN from MCN or as a malignancy marker, and recent publications raise doubts about its suitability [28,29]. Regarding glucose, the same meta-analysis attributed a sensitivity and specificity of 93% and 89% for M-PCLs, with an accuracy of 89% and an AUC of between 0.87 and 0.91 [30], surpassing CEA [31]. However, it is not a malignancy marker either, and reading errors occur in up to 22% of cases due to ICF viscosity [32]. There is also evidence of confounding results in PCs [33], with nearly 30% false positives. In the study cohort, the diagnostic performance of glucose was markedly lower, although the small number of cysts for which we have glucose values must be considered.

Regarding genomics in ICF, the identification of *KRAS* mutations showed high specificity (96%) and low sensitivity (45%) for IPMNs, as they may also exist in up to 35% of MCNs. The associations of *KRAS*-*GNAS* mutations result in higher sensitivity (91%), but their simultaneous presence is less than 50% [34]. Moreover, genomics is expensive and not widely available, as in our case.

There are other new techniques like needle-based confocal laser endomicroscopy, EUS through the needle biopsy, and contrast-enhanced EUS. Needle-based confocal laser endomicroscopy is a technique that provides high-resolution images by introducing a fiber optic probe (0.85 mm) through a 19G needle, allowing histological analysis of the cyst wall in real time. In different clinical trials, better performance with respect to CEA, and even EUS-FNA, has been observed in the characterization of PCLs, with a diagnostic yield of up to 88% and a diagnostic accuracy of up to 96%. However, it is a technique that is not free of complications, such as the increased risk of pancreatitis or intracystic hemorrhage. Other disadvantages are its high cost, which prolongs the examination time, the endoscopist’s learning curve, its limited availability, the technical difficulties preventing the acquisition of adequate images in certain situations, and the fact that it is not an optimal test for ruling out malignancies because it only allows the visualization of a small surface of the interior of the cyst [35]. EUS through the needle biopsy consists of introducing a mini-forceplier (Moray microforceplier) through a 19G needle to perform a direct biopsy of the cyst wall. Various publications have proven its usefulness in the characterization of PCLs, with an interesting diagnostic yield of around 62% for differentiating mucinous and non-mucinous lesions and up to 71.5% for detecting malignancies. Its main drawback is its significant association with adverse effects of up to 16% (11.5% in more recent series), the most frequent being intracystic hemorrhage. In a recently published series, risk predictors associated with complications were identified in the following order from highest to lowest: diagnosis of IPMN, number of passes (higher risk with more passes), age (higher risk with older age), and complete aspiration of the cyst. Thus, a high-risk group was identified (associated with 28% of adverse events), made up of patients diagnosed with IPMN in whom multiple passes were performed. Therefore, this is a technique in which it is essential to select the patient appropriately to ensure that the risk/benefit ratio is adequate, and this circumstance further limits its diagnostic yield [36]. Contrast-enhanced EUS involves using ultrasound contrasts simultaneously with EUS. These contrasts provide information on the microvascularization of the lesions, improving their characterization. In general, it is a safe technique with a very low rate of adverse effects. Most studies do not focus so much on its ability to characterize the different PCLs, but on its usefulness in characterizing mural nodules with suspected malignancies. A recent systematic review that analyzed eight studies in which contrast-enhanced EUS was performed concluded that the technique has a diagnostic accuracy of 95% in identifying malignant nodules [37]. However, its capacity for differential diagnosis of different PCLs is more limited, and it is not available in many centers, as in our case.

Faced with these results, the iTLB1 model showed sensitivity, specificity, accuracy, and AUC of 92%, 60%, 77%, and 0.79 for differentiating M-PCLs and NM-PCLs. Compared to other tools in the cohort (Table 3), it had superior sensitivity and NPV, and compared to data reported in the literature, its accuracy and AUC are only surpassed by glucose and genomics, but its results are not affected by ICF viscosity, and it has better affordability.

In the subsequent validation with lesions without a confirmed diagnosis, four PCs were misclassified (5/11 in total, only one confirmed histopathologically), all associated with severe underlying inflammation (advanced chronic pancreatitis or necrohemorrhagic acute pancreatitis), most likely reflecting the heterogeneous ICF composition of PCs due to its inflammatory pathophysiology. Presumably, by increasing the sample size, TLB models could not only identify this inflammatory nature but also potential complications, such as necrosis, infection, or hemorrhage, through the global analysis of ICF components. Evidence of this is the characteristic pattern of walled-off necrosis lesion thermogram already observed in our cohort (Figure 1).

### 4.2. Identification of Malignancies

Regarding distinguishing bM-PCL from mM-PCL, a recent meta-analysis [24] describes a sensitivity and specificity of 36–71% and 64–100% for MDCT, and 76% and 80% for MRI, with an accuracy of 81% when combining both [38]. In the study cohort, MDCT showed a higher sensitivity (88%) and specificity (88%), and MRI showed lower sensitivity (33%) but excellent specificity (100%), with EUS being the most accurate test (91%), with a sensitivity and specificity of 75% and 100%.

As shown in Table 3, HRS/WF showed low sensitivity but high specificity (up to 100% for some signs like solid components or suspicious lymphadenopathies) despite the performance being lower than described in consensus guidelines [25]. For these signs, the literature describes a limited isolated value on malignancy prediction, with increasing stepwise risk as the number of WF increases (22% with one, 100% with ≥4) [23]. In this study, both observations were observed, with examples of isolated mural nodules described in PCs, as well as a tendency for mM-PCLs to accumulate several HRS/WF (37% with ≥3). However, contrary scenarios were also observed, with M-PCLs with ≥3 WF without evidence of malignancy.

Two clinical signs were of particular relevance: new-onset diabetes (37% mM-PCLs), but a distracting factor in the surgical indication on two SCAs, and jaundice (50% mM-PCLs, 75% in the pancreatic head and in any NM-PCL or bM- PCL).

According to previous reviews [39], cytology showed high specificity (83–100%) but low sensitivity (50%), with up to 60% hypocellular samples. In this study, 20% of the samples were hypocellular, and existing malignancy was misdiagnosed in 20% of the cases.

Genomics in ICF can also detect malignancy using helpful mutations (*TP53*, *SMAD4*, *CDKN2A*, *PIK3CA*) with high specificity (92–98%) but low sensitivity (9–39%) [27] despite its cost and limited availability, as is the case for our center.

Faced with these data, the iTLB2 model was shown to be the most valuable tool in detecting malignancies, with a performance of 100%. Notably, it correctly identified one mM-PCL in its earliest stage of malignancy (HGD) that had been operated by growth, without any other WF. It must be noted that, given the small sample size, all M-PCLs (histopathologically confirmed or not) were included in the model training, so it could not be validated and could only be considered a pilot study. More studies are necessary to confirm these results.

Finally, a sub-analysis of surgical indications and complications was performed to assess the possible influence of both models on decision-making and patient outcomes. All surgeries were correctly indicated following current guidelines based on suspicion of mucinous origin and the presence of symptoms or HRS/WF. However, malignancy was only confirmed in 1/5 distal pancreatectomies (all of them presenting minor post-surgical complications) and in 3/5 CDPs (one post-surgical death), with the other two being SCAs (fortunately without post-surgical complications). In the hypothetical scenario where iTLB had been considered in decision-making, the iTLB1 model would not have identified the non-mucinous origin of the two operated SCAs, but the iTLB2 model would have correctly identified the absence of malignancies in the four benign distal pancreatectomies, avoiding surgery (67% of distal pancreatectomies) and its associated morbidity (minor complications).

Regarding limitations, this is a retrospective single-center study with a small sample size, with only 63% of histologically confirmed diagnoses; however, the diagnostic approach without histology follows current standards of clinical practice. ICF glucose determination was not performed in older samples because of the relatively recent evidence of its utility. The inclusion bias in patient recruitment was already commented on (for example, it would be very interesting to be able to assess the performance of this technique in asymptomatic patients, thus removing the selection bias in our study). TLB is a highly sensitive but less specific tool. TLB does not specifically inform on the identity of the proteins responsible for the changes observed in the thermogram and neither can the temperature pairs of the models be justified by biomarkers or biological mechanisms by themselves.

The continuation of this study will focus on increasing the sample size through multicenter studies, including additional glucose measurement and performing proteomics analysis to discover the proteins responsible for the thermogram patterns.

## 5. Conclusions 

Despite all these findings requiring future clinical validation through multicenter studies with larger sample sizes, iTLB emerges as a promising, minimally invasive, inexpensive, and easy-to-apply tool for improving differential diagnosis of PCLs and malignancy detection, even in the early stages. Its methodology allows the overall screening of the total composition of ICF in a single analytical process, avoiding the loss of information inherent in current strategies that consider individual biomarkers.

The future will probably bring diagnostic models that combine different tools using simultaneous analysis of different sources to increase diagnostic accuracy. iTLB has this multiple analysis strategy as one of its best advantages, and therefore, in our opinion, it could be a key diagnostic method.

## Figures and Tables

**Table 1:** Classification of pancreatic cystic lesions.

Non-Mucinous PCLs (NM-PCLs).	Mucinous PCLs (M-PCLs)
Pseudocyst (PC) Serous cystadenoma (SCA) Lymphoepithelial cyst/lymphangioma (LINF)	MD-IPMN (main duct IPMN) BD-IPMN (branch duct IPMN) Mucinous cystic neoplasm (MCN) Simple mucinous cyst (SMC)
	Further subdivided into: Benign (bM-PCL) Malignant (mM-PCL): ○ Malignant IPMN (mIPMN) ○ PDAC with cystic component (cPDAC). ○ SMC with high-grade dysplasia (SMC-HGD).

Malignant pancreatic cystic lesions were pathologically confirmed.

**Table 2:** Descriptive characteristics of the cohort of patients with pancreatic cystic lesions (PCLs), including non-mucinous PCLs (NM-PCLs) and mucinous PCLs (M-PCLs).

Cyst #	n	% Total (% Group)	Age *	Sex: n (%) ♀	Size ** (mm)	Loc: n (%) B-T ***
NM-PCLs:	18	51% (100%)	64 (49–86)	8 (44%)	46 [38; 62]	8 (44%)
PC	11	31% (61%)	62 (49–75)	3 (27%)	55 [33; 85]	5 (45%)
SCA	5	14% (28%)	72 (60–86)	4 (80%)	45 [40; 45]	2 (40%)
LINF	2	6% (11%)	55 (52–59)	1 (50%)	48 [47; 48]	1 (50%)
M-PCLs:	17	49% (100%)	61 (36–83)	10 (59%)	32 [26; 50]	9 (52%)
IPMN	10	29% (59%)	68 (48–83)	4 (40%)	28 [25; 35]	4 (40%)
MCN	2	6% (12%)	39 (36–42)	2 (100%)	31 [30; 31]	2 (100%)
SMC-HGD	1	3% (6%)	55 (55–55)	1 (100%)	58 [58; 58]	1 (100%)
PDAC	4	11% (23%)	53 (38–74)	3 (75%)	65 [44; 80]	2 (50%)
Total	35	100%	62 (36–86)	18 (51%)	40 [30; 52]	17 (49%)

Data are categorized by cyst type, number of cases (n), percentage of total cases, age *, sex distribution, size of the lesions **, and their location ***. * Mean age of patients with the minimum and maximum in parentheses. ** Median size of the lesions in millimeters with the interquartile range in brackets. *** Number and percentage of lesions located in the body-tail (B-T) of the pancreas. Cyst #: Type of pancreatic cystic lesion, PC: Pseudocyst, SCA: Serous Cystadenoma, LINF: Lymphoepithelial cyst/Lymphangioma, IPMN: Intraductal Papillary Mucinous Neoplasm, MCN: Mucinous Cystic Neoplasm, SMC-HGD: Simple Mucinous Cyst with High-Grade Dysplasia, PDAC: Pancreatic Ductal Adenocarcinoma.

**Table 3:** Diagnostic performance of imaging tests, HRS/WF, string sign, biochemical markers, and classification models in differentiating non-mucinous vs. mucinous and benign vs. malignant pancreatic cystic lesions (PCLs).

	Non-Mucinous vs. Mucinous					Benign vs. Malignant				
	Acc	Sens	Spec	PPV	NPV	Acc	Sens	Spec	PPV	NPV
MDCT	75%	73%	80%	89%	57%	88%	88%	88%	88%	88%
MRI	56%	44%	71%	67%	50%	75%	33%	100%	100%	71%
EUS	77%	67%	90%	89%	69%	91%	75%	100%	100%	88%
Dilated Wirsung						65%	50%	78%	67%	64%
Nodules						53%	12%	89%	50%	53%
Solid component						76%	50%	100%	100%	69%
Lymph						65%	25%	100%	100%	60%
String sign (ICF)	71%	59%	83%	77%	68%					
ICF-CEA	79%	76%	82%	81%	78%					
ICF Glucose	60%	50%	67%	50%	67%					
Serum Ca19.9						68%	62%	70%	45%	82%
iTLB1 Model	77%	92%	60%	73%	86%					
iTLB2 Model						100%	100%	100%	100%	100%

The metrics include accuracy (Acc), sensitivity (Sens), specificity (Spec), positive predictive value (PPV), and negative predictive value (NPV). Each column under “Non-Mucinous vs. Mucinous” and “Benign vs. Malignant” provides the respective performance metrics (Acc, Sens, Spec, PPV, NPV) for the listed diagnostic tools and models. Imaging Studies: Multidetector Computed Tomography (MDCT), Magnetic Resonance Imaging (MRI), Endoscopic Ultrasound (EUS), presence of a dilated main pancreatic duct (Dilated Wirsung), presence of mural nodules (Nodules), presence of a solid component within the cyst (Solid Component), and presence of suspicious lymphadenopathies (Lymph). Intracystic fluid Characteristics: presence of a string sign in ICF indicating mucinous content (String Sign [ICF]). Biochemical Markers: Carcinoembryonic Antigen (ICF-CEA) in ICF, glucose level in ICF (ICF Glucose), and Carbohydrate Antigen 19.9 in serum (serum Ca19.9). Classification Models: iTLB1 Model, which differentiates non-mucinous vs. mucinous PCLs, and iTLB2 Model, which differentiates benign vs. malignant mucinous PCLs. **Notes**: Thresholds for CEA, glucose, and Ca19.9 are 192 ng/mL, 50 mg/dL, and 37 U/mL, respectively; iTLB: intelligence Thermal Liquid Biopsy.

## Data Availability

The data presented in this study are available on request from the corresponding author.

## Supplementary Materials

The following supporting information can be downloaded at: https://www.mdpi.com/article/10.3390/cancers16234024/s1, Tables S1 and S2: Descriptive characteristics and clinical data for patients with pancreatic cystic lesions (PCLs). This table presents the descriptive characteristics and clinical data of patients diagnosed with pancreatic cystic lesions (PCLs), including non-mucinous PCLs (NM-PCLs) and mucinous PCLs (M-PCLs). Data are organized by patient ID, lesion location (LOC), size of the lesion, clinical context, intracystic fluid (ICF) characteristics, presence of Worrisome features (WF) or Wirsung communication, biochemical markers in ICF (BQ markers ICF), serum markers, symptoms, surgery details, diagnosis, other relevant information, and histological confirmation.

## References

[1] Michl P., Löhr M., Neoptolemos J.P., Capurso G., Rebours V., Malats N., Ollivier M., Ricciardiello L. (2021). UEG Position Paper on Pancreatic Cancer. Bringing Pancreatic Cancer to the 21st Century: Prevent, Detect, and Treat the Disease Earlier and Better. United Eur. Gastroenterol. J..

[2] Ergenc M., Karpuz S., Ergenc M., Yegen C. (2021). Enhanced Recovery after Pancreatic Surgery: A Prospective Randomized Controlled Clinical Trial. J. Surg. Oncol..

[3] Privalov G., Kavina V., Freire E., Privalov P.L. (1995). Precise Scanning Calorimeter for Studying Thermal Properties of Biological Macromolecules in Dilute Solution. Anal. Biochem..

[4] Garbett N.C., Brock G.N. (2016). Differential Scanning Calorimetry as a Complementary Diagnostic Tool for the Evaluation of Biological Samples. Biochim. Biophys. Acta Gen. Subj..

[5] Dandé, Nöt L.G., Wiegand N., Kocsis B., Lőrinczy D. (2017). DSC Analysis of Human Synovial Fluid Samples in the Diagnostics of Non-Septic and Septic Arthritis. J. Therm. Anal. Calorim..

[6] Chagovetz A.A., Jensen R.L., Recht L., Glantz M., Chagovetz A.M. (2011). Preliminary Use of Differential Scanning Calorimetry of Cerebrospinal Fluid for the Diagnosis of Glioblastoma Multiforme. J. Neurooncol..

[7] Pultrone L., Schmid R., Waltimo T., Braissant O., Astasov-Frauenhoffer M. (2022). Saliva Profiling with Differential Scanning Calorimetry: A Feasibility Study with Ex Vivo Samples. PLoS ONE.

[8] Velazquez-Campoy A., Vega S., Sanchez-Gracia O., Lanas A., Rodrigo A., Kaliappan A., Hall M.B., Nguyen T.Q., Brock G.N., Chesney J.A. (2018). Thermal Liquid Biopsy for Monitoring Melanoma Patients under Surveillance during Treatment: A Pilot Study. Biochim. Biophys. Acta Gen. Subj..

[9] Annesi F., Hermoso-Durán S., Rizzuti B., Bruno R., Pirritano D., Petrone A., Del Giudice F., Ojeda J., Vega S., Sanchez-Gracia O. (2021). Thermal Liquid Biopsy (TLB) of Blood Plasma as a Potential Tool to Help in the Early Diagnosis of Multiple Sclerosis. J. Pers. Med..

[10] Vega S., Garcia-Gonzalez M.A., Lanas A., Velazquez-Campoy A., Abian O. (2015). Deconvolution Analysis for Classifying Gastric Adenocarcinoma Patients Based on Differential Scanning Calorimetry Serum Thermograms. Sci. Rep..

[11] Rodrigo A., Ojeda J.L., Vega S., Sanchez-Gracia O., Lanas A., Isla D., Velazquez-Campoy A., Abian O. (2019). Thermal Liquid Biopsy (TLB): A Predictive Score Derived from Serum Thermograms as a Clinical Tool for Screening Lung Cancer Patients. Cancers.

[12] Hermoso-Durán S., Fraunhoffer N., Millastre-Bocos J., Sanchez-Gracia O., Garrido P.F., Vega S., Lanas Á., Iovanna J., Velázquez-Campoy A., Abian O. (2024). Development of a Machine-Learning Model for Diagnosis of Pancreatic Cancer from Serum Samples Analyzed by Thermal Liquid Biopsy. Adv. Intell. Syst..

[13] Hermoso-Durán S., García-Rayado G., Ceballos-Laita L., Sostres C., Vega S., Millastre J., Sánchez-Gracia O., Ojeda J.L., Lanas Á., Velázquez-Campoy A. (2021). Thermal Liquid Biopsy (TLB) Focused on Benign and Premalignant Pancreatic Cyst Diagnosis. J. Pers. Med..

[14] Téoule P., Bartel F., Birgin E., Rückert F., Wilhelm T.J. (2019). The Clavien-Dindo Classification in Pancreatic Surgery: A Clinical and Economic Validation. J. Investig. Surg..

[15] Rahib L., Wehner M.R., Matrisian L.M., Nead K.T. (2021). Estimated Projection of US Cancer Incidence and Death to 2040. JAMA Netw. Open.

[16] Grossberg A.J., Chu L.C., Deig C.R., Fishman E.K., Hwang W.L., Maitra A., Marks D.L., Mehta A., Nabavizadeh N., Simeone D.M. (2020). Multidisciplinary Standards of Care and Recent Progress in Pancreatic Ductal Adenocarcinoma. CA Cancer J. Clin..

[17] Goggins M., Overbeek K.A., Brand R., Syngal S., Del Chiaro M., Bartsch D.K., Bassi C., Carrato A., Farrell J., Fishman E.K. (2020). Management of Patients with Increased Risk for Familial Pancreatic Cancer: Updated Recommendations from the International Cancer of the Pancreas Screening (CAPS) Consortium. Gut.

[18] Chen W., Ahmed N., Krishna S.G. (2022). Pancreatic Cystic Lesions: A Focused Review on Cyst Clinicopathological Features and Advanced Diagnostics. Diagnostics.

[19] Vilela A., Quingalahua E., Vargas A., Hawa F., Shannon C., Carpenter E.S., Shi J., Krishna S.G., Lee U.-J., Chalhoub J.M. (2024). Global Prevalence of Pancreatic Cystic Lesions in the General Population on Magnetic Resonance Imaging: A Systematic Review and Meta-Analysis. Clin. Gastroenterol. Hepatol..

[20] Aziz H., Acher A.W., Krishna S.G., Cloyd J.M., Pawlik T.M. (2022). Comparison of Society Guidelines for the Management and Surveillance of Pancreatic Cysts. JAMA Surg..

[21] Valsangkar N.P., Morales-Oyarvide V., Thayer S.P., Ferrone C.R., Wargo J.A., Warshaw A.L., Fernández-del Castillo C. (2012). 851 Resected Cystic Tumors of the Pancreas: A 33-Year Experience at the Massachusetts General Hospital. Surgery.

[22] Schechter S., Shi J. (2017). Simple Mucinous Cyst of the Pancreas: Review and Update. Arch. Pathol. Lab. Med..

[23] Ohtsuka T., Fernandez-del Castillo C., Furukawa T., Hijioka S., Jang J.-Y., Lennon A.M., Miyasaka Y., Ohno E., Salvia R., Wolfgang C.L. (2024). International Evidence-Based Kyoto Guidelines for the Management of Intraductal Papillary Mucinous Neoplasm of the Pancreas. Pancreatology.

[24] Abraham A.S., Simon B., Eapen A., Sathyakumar K., Chandramohan A., Raju R.S., Joseph P., Kodiatte T.A., Gowri M. (2020). Role of Cross-Sectional Imaging (CT/MRI) in Characterization and Distinguishing Benign from Malignant/Potentially Malignant Cystic Lesions of Pancreas. J. Clin. Imaging Sci..

[25] European Study Group on Cystic Tumours of the Pancreas (2018). European Evidence-Based Guidelines on Pancreatic Cystic Neoplasms. Gut.

[26] Bick B., Enders F., Levy M., Zhang L., Henry M., Dayyeh B., Chari S., Clain J., Farnell M., Gleeson F. (2015). The String Sign for Diagnosis of Mucinous Pancreatic Cysts. Endoscopy.

[27] Pflüger M.J., Jamouss K.T., Afghani E., Lim S.J., Rodriguez Franco S., Mayo H., Spann M., Wang H., Singhi A., Lennon A.M. (2023). Predictive Ability of Pancreatic Cyst Fluid Biomarkers: A Systematic Review and Meta-Analysis. Pancreatology.

[28] Gaddam S., Ge P.S., Keach J.W., Mullady D., Fukami N., Edmundowicz S.A., Azar R.R., Shah R.J., Murad F.M., Kushnir V.M. (2015). Suboptimal Accuracy of Carcinoembryonic Antigen in Differentiation of Mucinous and Nonmucinous Pancreatic Cysts: Results of a Large Multicenter Study. Gastrointest. Endosc..

[29] Kwan M.C., Pitman M.B., Fernandez-del Castillo C., Zhang M.L. (2024). Revisiting the Performance of Cyst Fluid Carcinoembryonic Antigen as a Diagnostic Marker for Pancreatic Mucinous Cysts: A Comprehensive 20-Year Institutional Review. Gut.

[30] Ribeiro T., Lopes S., Moutinho-Ribeiro P., Macedo G., Vilas-Boas F. (2024). Performance of Intracystic Glucose Measurement for the Characterization of Pancreatic Cystic Lesions. J. Gastrointest. Liver Dis..

[31] Smith Z.L., Satyavada S., Simons-Linares R., Mok S.R.S., Martinez Moreno B., Aparicio J.R., Chahal P. (2022). Intracystic Glucose and Carcinoembryonic Antigen in Differentiating Histologically Confirmed Pancreatic Mucinous Neoplastic Cysts. Am. J. Gastroenterol..

[32] Noia J.L., Mejuto R., Oria I., De la Iglesia-García D., Villaverde A., Voces A., Pizzala J., Iglesias-García J., Urgiles D., Marcolongo M. (2022). Rapid Diagnosis of Mucinous Cystic Pancreatic Lesions by On-Site Cyst Fluid Glucometry. Surg. Endosc..

[33] Carr R.A., Yip-Schneider M.T., Simpson R.E., Dolejs S., Schneider J.G., Wu H., Ceppa E.P., Park W., Schmidt C.M. (2018). Pancreatic Cyst Fluid Glucose: Rapid, Inexpensive, and Accurate Diagnosis of Mucinous Pancreatic Cysts. Surgery.

[34] McCarty T.R., Paleti S., Rustagi T. (2021). Molecular Analysis of EUS-Acquired Pancreatic Cyst Fluid for KRAS and GNAS Mutations for Diagnosis of Intraductal Papillary Mucinous Neoplasia and Mucinous Cystic Lesions: A Systematic Review and Meta-Analysis. Gastrointest. Endosc..

[35] Facciorusso A., Buccino V.R., Sacco R. (2020). Needle-Based Confocal Laser Endomicroscopy in Pancreatic Cysts: A Meta-Analysis. Eur. J. Gastroenterol. Hepatol..

[36] Facciorusso A., Kovacevic B., Yang D., Vilas-Boas F., Martínez-Moreno B., Stigliano S., Rizzatti G., Sacco M., Arevalo-Mora M., Villarreal-Sanchez L. (2022). Predictors of Adverse Events after Endoscopic Ultrasound-Guided through-the-Needle Biopsy of Pancreatic Cysts: A Recursive Partitioning Analysis. Endoscopy.

[37] Lisotti A., Napoleon B., Facciorusso A., Cominardi A., Crinò S.F., Brighi N., Gincul R., Kitano M., Yamashita Y., Marchegiani G. (2021). Contrast-Enhanced EUS for the Characterization of Mural Nodules within Pancreatic Cystic Neoplasms: Systematic Review and Meta-Analysis. Gastrointest. Endosc..

[38] Udare A., Agarwal M., Alabousi M., McInnes M., Rubino J.G., Marcaccio M., van der Pol C.B. (2021). Diagnostic Accuracy of MRI for Differentiation of Benign and Malignant Pancreatic Cystic Lesions Compared to CT and Endoscopic Ultrasound: Systematic Review and Meta-analysis. J. Magn. Reson. Imaging.

[39] Gillis A., Cipollone I., Cousins G., Conlon K. (2015). Does EUSFNA Molecular Analysis Carry Additional Value When Compared to Cytology in the Diagnosis of Pancreatic Cystic Neoplasm? A Systematic Review. HPB.

