# Supplementary material for: Thermal Liquid Biopsy: A Promising Tool for the Differential Diagnosis of Pancreatic Cystic Lesions and Malignancy Detection
Source: Cancers (Basel). 2024 Nov 30;16(23):4024. doi: 10.3390/cancers16234024 (PMC11640424; doi:10.3390/cancers16234024)
Supplement: Supplementary file 1 [file cancers-16-04024-s001.zip › cancers-3314202-supplementary.pdf]

SUPPLEMENTARY MATERIAL (Table S1 and S2)

	LOC	SIZE (mm)	Clinical Context	ICF	WF/Wirsung communication	BQ-markers ICF	Serum markers	Symptoms	Surgery	Diagnose	Other relevant information	Histological confirmation
**Q1**	Body	50	♂ 72y	Serohematic	No/No	CEA = 0.69 ng/mL<br>Amylase = 41 U/L	Brb T = 0.65 mg/dL<br>**CA 19.9 = 45.7 U/mL**	-	-	SCA	**Pathognomonic morphology**<br>Post-puncture intracystic bleeding	
**Q4**	Body	55	♂ 49y<br>-Alcohol and Tobacco<br>-Chronic Pancreatitis	Brownish with debris (**WON**)	No/No	CEA = 9.23 ng/mL<br>Amylase = 29272 U/L	Brb T = 0.12 mg/dL<br>CA 19.9 = 20 U/mL	Abdominal pain	YES	PC (WON)	Pancreatic Fistula secondary to abdominal aortic surgery	✓<br>Surgical specimen
**Q10**	Tail	64	♀ 72y DM<br>-Chronic Pancreatitis	Brownish with debris (**WON**))	No/No	CEA = 2.45 ng/mL<br>Amylase = 30926 U/L	Brb T = 0.32 mg/dL<br>CA 19.9 = 8.59 U/mL	Abdominal pain	-	PC (WON)	Involution in image control (TC)<br>Ig G 4 +	
**Q12**	Head	49	♀ 52y DM	Chylous	No/No	CEA = 0.78 ng/mL<br>Amylase = 24 U/L	Brb T = 0.52 mg/dL		-	LINF	Lymphoepithelial cyst: plenty of mature lymphoid elements and follicles in ICF	✓<br>Cytology
**Q13**	Head	100	♂ 63y DM<br>-Alcohol and Tobacco<br>-Chronic Pancreatitis	Brownish	Wirsung 10mm*/Yes	CEA = 68,4 ng/mL<br>Amylase = 5635 U/L	Brb T = 0.5 mg/dL<br>CA 19.9 = 29.6 U/mL	Abdominal pain	YES	PC	Duct disconnection syndrome	✓<br>Surgical specimen
**Q15**	Head	100	♂ 59y<br>-Chronic Pancreatitis	Brownish with debris (**WON**)	No/No	CEA = 1.99 ng/mL<br>Amylase = 53159 U/L	Brb T = 0.45 mg/dL	Abdominal pain	YES	PC (WON)	Urgent hemoperitoneum surgery	✓<br>Surgical specimen
**Q16**	Body	40	♂ 54y<br>-Alcohol<br>-**Severe acute pancreatitis**	Brownish	No/No	CEA = 59 ng/mL<br>Amylase > 11000 U/L	Brb T = 0.3 mg/dL	Abdominal pain	-	PC	Involution in image control (TC)	
**Q18**	Body	150	♀ 71y	Brownish with debris (**WON**)	No/No	-	Brb T = 0.3 mg/dL	Abdominal pain	YES	PC (WON)		✓<br>Surgical specimen
**Q20**	Head	30	♂ 58y DM<br>-Alcohol and Tobacco<br>-Chronic Pancreatitis	Transparent<br>**String sign**	**Solid Wall Nodule 8mm**. Dilated and irregular Wirsung*/No	CEA = 28.8 ng/mL<br>Amylase > 50000 U/L	Brb T = 0.5 mg/dL<br>CA 19.9 = 10.1 U/mL	Abdominal pain	-	PC	Involution in image control (TC + USE)<br>Cytology: proteinaceous material and isolated inflammatory cells	
**Q22**	Head	45	♀ 70y **New Onset DM**	Serohematic<br>Non-Filant	**Thickened walls** /No	CEA = 377 ng/mL<br>Amylase = 835 U/L	Brb T = 0.24 mg/dL<br>CA 19.9 = 29.7 U/mL	-	YES	SCA	Post-puncture intracystic bleeding	✓<br>Surgical specimen

**Q26**	Body	47	♂ 59y	Chylous	No/No	CEA = 0.3 ng/mL<br>Glc = 68 mg/dL<br>Amylase = 29 U/L	Brb T = 0.5 mg/dL<br>CA 19.9 = 8.83 U/mL	-	-	LINF	Lymphangioma: High triglycerides in ICF<br>Retroperitoneal. No changes in TC control	✓<br>Cytology
**Q27**	Head	45	♀ 73y	Serous	No/No	CEA = 47 ng/mL<br>Glc = 107 mg/dL<br>Amylase = 43 U/L	Brb T = 0.5 mg/dL<br>CA 19.9 = 0.6 U/mL	-	-	SCA	**Pathognomonic morphology**<br>No changes in image control (TC)	
**Q28**	Head	40	♀ 60y **New onset DM**<br>-Tobacco	Transparent<br>**String sign**	No/Yes	CEA = 93 ng/mL<br>Glc = 117 mg/dL<br>Amylase > 86000 U/L	Brb T = 0.29 mg/dL<br>**CA 19.9 = 111 U/mL**	Abdominal pain	YES	SCA		✓<br>Surgical specimen
**Q32**	Body	29	♀ 86y DM	Serous	No/Yes (RM)	CEA = 25 ng/mL<br>Glc = 0 mg/dL<br>Amylase = 2 U/L	Brb T = 0.8 mg/dL	Abdominal pain	-	SCA	No changes in image control (ECO)	
**Q33**	Body	29	♂ 52y<br>-Alcohol and Tobacco<br>- Chronic Pancreatitis	Serohematic<br>**String sign**	No/No	CEA = 91.7 ng/mL<br>Amylase = 114800 U/L	Brb T = 0.47 mg/dL	Abdominal pain	-	PC	No changes in image control (TC)	
**Q37**	Head	37	♀ 61y<br>-Chronic Pancreatitis	Brownish	Dilated and irregular Wirsung, Thickened walls*/No	CEA = 3186 ng/mL<br>Amylase = 12160 U/L	Brb T = 0.39 mg/dL<br>**CA 19.9 = 81.9 U/mL**	Abdominal pain	-	PC	Involution in image control (RM)<br>Cytology: debris and acute inflammation	
**Q40**	Head	30	♂ 69y<br>-Alcohol and Tobacco<br>- Chronic Pancreatitis	Brownish	Dilated and irregular Wirsung, Thickened walls */No	CEA = 92 ng/mL<br>Glc = 42 mg/dL<br>Amylase = 54336 U/L	Brb T = 0.2 mg/dL<br>CA 19.9 = 18.2 U/mL	Abdominal pain<br>Weight loss	-	PC	Involution in image control (ECO)	
**Q41**	Head	70	♂ 75y DM<br>- Tobacco	Brownish	No/No	CEA = 225 ng/mL<br>Glc = 60 mg/dL<br>Amylase = 27640 U/L	Brb T = 0.7 mg/dL<br>**CA 19.9 = 76.3 U/mL**	-	-	PC	Involution in image control (RM)	

*In the context of chronic pancreatitis

*In the context of chronic pancreatitis

ID	LOC	SIZE (mm)	Clinical Context	ICF	WF/Wirsung communication	BQ-markers ICF	Serum markers	Symptoms	Surgery	Diagnose	Other relevant information	Histological confirmation
**Q2**	Body	26	♂ 74y<br>-Alcohol and Tobacco	Transparent<br>**String sign**	No/No	CEA = 489 ng/mL<br>Amylase = 17337 U/L	Brb T = 0. 95 mg/dL<br>CA 19.9 = 2 U/mL	-	-	IPMN<br>(BD)	Multiple small cysts on control EUS, highly suggestive of IPMN	
**Q3**	Body	20	♀ 79y	Transparent<br>**String sign**	**Wirsung 5mm**/No	CEA = 1488 ng/mL<br>Amylase = 162 U/L	Brb T = 0. 45 mg/dL<br>CA 19.9 = 12.47 U/mL	-	-	IPMN<br>(mixed)	Multiple small cysts. No changes in image control (USE)	
**Q5**	Body	32	♀ 42y	Transparent<br>**String sign**	No/No	CEA = 1617 ng/mL<br>Amylase = 3401 U/L	Brb T = 0. 68 mg/dL<br>CA 19.9 = 24.4 U/mL	Abdominal pain	YES	MCN		✓<br>Surgical specimen
**Q7**	Head	25	♂ 76y DM	Transparent<br>**String sign**	No/Yes	CEA = 156 ng/mL<br>Amylase = 56593 U/L	Brb T = 0.5 mg/dL	-	-	IPMN<br>(BD)	Multiple small cysts. RCI hemodialysis	
**Q8**	Head	25	♂ 72y<br>**-Acute pancreatitis**	Transparent<br>**String sign**	**Solid Wall Nodule 7mm**/ Yes	CEA = 556 ng/mL<br>Amylase = 28600 U/L	Brb T = 0.4 mg/dL<br>**CA 19.9 = 805 U/mL**	Abdominal pain	YES	IPMN<br>(BD)	Cytology; mucin-producing columnar cells, no atypia	✓<br>Surgical specimen
**Q9**	Head	30	♂ 53y<br>-Chronic Pancreatitis	Transparent<br>**String sign**	**Solid Wall Nodule 9mm, Wirsung 5mm**/No	CEA = 392 ng/mL<br>Amylase = 10 U/L	**Brb T = 9 mg/dL**<br>**CA 19.9 = 128 U/mL**	**Jaundice**	YES	IPMNm<br>(MD)		✓<br>Surgical specimen
**Q11**	Head	35	♀ 83y DM<br>-Chronic pancreatitis	Serohematic	**Wirsung 10mm**/ No	CEA > 50000 ng/mL<br>Amylase = 4 U/L	Brb T = 0.32 mg/dL<br>**CA 19.9 = 754 U/mL**	Diarrhea<br>Weight loss	-	IPMN<br>(MD)	Normal CEA<br>Negative cytology for malignancy	
**Q14**	Head	35	♂ 65y<br>-Alcohol and Tobacco	Transparent	No/No	CEA = 225 ng/mL<br>Amylase > 46000 U/L	Brb T = 0.39 mg/dL<br>CA 19.9 = 21.4 U/mL	Abdominal pain	YES	IPMN<br>(BD)		✓<br>Surgical specimen
**Q17**	Head	50	♀ 38y<br>-Tobacco<br>-Chronic Pancreatitis	Transparent<br>**String sign**	Wirsung 8mm (obstructive)/ No	CEA = 1192 ng/mL<br>Amylase > 13000 U/L	**Brb T = 13.15 mg/dL**<br>CA 19.9 = 2 U/mL	**Jaundice**<br>Weight loss	YES	PDACc	Peritoneal carcinomatosis	✓<br>Surgical specimen
**Q19**	Body-tail	80	♀ 42y<br>-Tobacco	Serohematic	**Solid component with cystic areas**/ No	CEA = 29.2 ng/mL<br>Amylase = 51 U/L	Brb T = 0.43 mg/dL<br>**CA 19.9 = 155 U/mL**	Weight loss		PDACc	T3N2M1	✓<br>USE-FNB
**Q25**	Body	50	♀ 69y **New onset DM**	Serohematic	**Solid component 30mm, Wirsung 7.2mm**/ Yes	CEA = 3011 ng/mL<br>Glc = 33 mg/dL<br>Amylase = 511 U/L	**Brb T = 11.27 mg/dL**<br>**CA 19.9 < 2* U/mL**	**Jaundice**<br>Weight loss	YES	IPMNm<br>(BD)		✓<br>Surgical specimen
**Q29**	Head	20	♂ 48y **New onset DM**<br>-Tobacco	Transparent<br>**String sign**	**Solid component, Dilated Wirsung,** /No	CEA = 621 ng/mL<br>Glc = 66 mg/dL<br>Amylase = 47827 U/L	**Brb T = 9 mg/dL**<br>**CA 19.9 = 113 U/mL**	**Jaundice**<br>Weight loss	YES	IPMNm<br>(mixed)		✓<br>Surgical specimen

**Q31**	Body	34	♀ 65y	Serohematic<br>**String sign**	Peripheral Calcification/Yes	CEA = 3 ng/mL<br>Glc = 91 mg/dL<br>Amylase = 323 U/L	Brb T = 0.33 mg/dL<br>CA 19.9 = 18.4 U/mL	-	-	IPMN	No changes in image control (RM)	
**Q34**	Body	80	♀ 74y	Transparent<br>**String sign**	**Solid component**/No	CEA = 14742 ng/mL<br>Glc = 2 mg/dL<br>Amylase = 110 U/L	Brb T = 0.5 mg/dL<br>**CA 19.9 = 8440 U/mL**	Abdominal pain<br>Weight loss	YES	PDACc	Peritoneal carcinomatosis	✓<br>Surgical specimen
**Q35**	Tail	30	♀ 36y	Transparent	No/No	CEA = 29,4 ng/mL<br>Amylase = 183000 U/L	Brb T = 0.44 mg/dL<br>CA 19.9 = 21.5 U/mL	Abdominal pain	YES	MCN		✓<br>Surgical specimen
**Q36**	Body-tail	58	♀ 55y	Brownish with debris	No/No	CEA =268921 ng/mL<br>Amylase = 34 U/L	Brb T = 0.55 mg/dL<br>CA 19.9 = 6.6 U/mL	-	YES	SMC with HGD	Flat mucinous epithelium without papillary architecture, NO Wirsung communication, NO ovaric stroma	✓<br>Surgical specimen
**Q38**	Head	27	♂ 59y **New onset DM**<br>-Alcohol<br>-Chronic Pancreatitis	Serohematic	**Wirsung 5mm, thickened walls**/No	CEA = 673 ng/mL<br>Amylase = 2928 U/L	Brb T = 0.35 mg/dL<br>**CA 19.9 = 51.5 U/mL**	Weight loss	YES	PDACc	T2N0M0	✓<br>Surgical specimen

Table S1 and S2: Descriptive Characteristics and Clinical Data of Patients with Pancreatic Cystic Lesions (PCL)

This table presents the descriptive characteristics and clinical data of patients diagnosed with Pancreatic Cystic Lesions (PCL), including Non-Mucinous PCL (NM-PCL) and Mucinous PCL (M-PCL). Data is organized by patient ID, lesion location (LOC), size of the lesion, clinical context, intracystic fluid (ICF) characteristics, presence of Worrisome Features (WF) or Wirsung communication, biochemical markers in ICF (BQ-markers ICF), serum markers, symptoms, surgery details, diagnosis, other relevant information, and histological confirmation.

- **LOC**: Location of the lesion within the pancreas (Head, Body, Tail).
- **SIZE (mm)**: Size of the lesion in millimeters.
- **Clinical Context**: Relevant patient history and clinical conditions, including comorbidities such as Diabetes Mellitus (DM), alcohol and tobacco use, and chronic pancreatitis.
- **ICF**: Characteristics of the intracystic fluid, including appearance and any specific markers like serohematic or chylous fluid.
- **WF/Wirsung communication**: Presence of Worrisome Features or communication with the pancreatic duct (Wirsung).
- **BQ-markers ICF**: Biochemical markers present in the intracystic fluid, including Carcinoembryonic Antigen (CEA), amylase levels, and glucose concentration (Glc).
- **Serum markers**: Serum levels of bilirubin (Brb T) and Carbohydrate Antigen 19-9 (CA 19.9).
- **Symptoms**: Clinical symptoms reported by patients, such as abdominal pain, jaundice, weight loss, and new onset of diabetes.
- **Surgery**: Indication of whether surgery was performed (Yes/No).
- **Diagnose**: Final diagnosis of the lesion type, including specific PCL subtypes like Pseudocyst (PC), Serous Cystadenoma (SCA), Lymphoepithelial Cyst (LINF), Intraductal Papillary Mucinous Neoplasm (IPMN), Mucinous Cystic Neoplasm (MCN), Simple

Mucinous Cyst with High-Grade Dysplasia (SMC-HGD), and Pancreatic Ductal Adenocarcinoma (PDAC).

- **Other relevant information**: Additional clinical details, such as pathognomonic morphology, post-puncture complications, involution in imaging control, and cytological findings.
- **Histological confirmation**: Indication of whether the diagnosis was confirmed histologically (Yes/No).
